# Supplementary material for: Temperature difference and systemic risk: Evidence from LASSO-VAR-DY based on China’s pan-financial market
Source: PLoS One. 2024 Mar 15;19(3):e0295575. doi: 10.1371/journal.pone.0295575 (PMC10942056; doi:10.1371/journal.pone.0295575)
Supplement: Attachment — Submitted filename: Response to Reviewers.docx [file pone.0295575.s002.docx]

Dear Academic Editor,

Thank you very much for your comments and professional advice. These opinions help to improve acade micrigor of our article Based on your suggestion and request, we have made corrected modifications on the revised manuscript. Meanwhile, the manuscript had be reviewed and edited by language and structure. We hope that our work can be improved again. Furthermore,we would like to show the details as follows:

**Submit Requirements:**

**Comment1#**

“ Please include the following items when submitting your revised manuscript: 'Response to Reviewers','Revised Manuscript with Track Changes','Manuscript'. ”

**The author's answer:** We have followed the process associated with the PLOS ONE submission system by submitting the above items. Each file has been uploaded separately. In flie 'Revised Manuscript with Track Changes', We have highlighted the changes in red.

**Journal Requirements:**

**Comment1#**

“ 1. Please ensure that your manuscript meets PLOS ONE's style requirements, including those for file naming. The PLOS ONE style templates can be found at https://journals.plos.org/plosone/s/file?id=wjVg/PLOSOne_formatting_sample_main_body.pdf and https://journals.plos.org/plosone/s/file?id=ba62/PLOSOne_formatting_sample_title_authors_affiliations.pdf. ”

**The author's answer:**We have revised the manuscript to conform to PLOS ONE style requirements, including file naming requirements.In the process, files PLOSOne_formatting_sample_main_body.pdf and PLOSOne_formatting_sample_title_authors_affiliations.pdf were also downloaded and revised according to their specific requirements.

**Comment2#**

“ 2. In your Data Availability statement, you have not specified where the minimal data set underlying the results described in your manuscript can be found. PLOS defines a study's minimal data set as the underlying data used to reach the conclusions drawn in the manuscript and any additional data required to replicate the reported study findings in their entirety. All PLOS journals require that the minimal data set be made fully available. For more information about our data policy, please see http://journals.plos.org/plosone/s/data-availability. ”

**The author's answer:**We have refined the submitted data in accordance with the Data Availability Statement. The underlying data used to reach the conclusions drawn in the manuscript and any additional data required to replicate the reported study findings in their entirety were packaged into minimal data sets. We named the file “Appendix. Data” and uploaded it to the submission system as Supporting Information.

**Comment3#**

“ 3. Please include captions for your Supporting Information files at the end of your manuscript, and update any in-text citations to match accordingly. Please see our Supporting Information guidelines for more information: [http://journals.plos.org/plosone/s/supporting-information. ”](http://journals.plos.org/plosone/s/supporting-information.\”)

**The author's answer:**We have referred to the Supporting Information guidelines and included the title of the Supporting Information document at the end of our manuscript. The details as follows:“Supporting information S1 Appendix. Data file”.

**Additional Editor Comments:**

**Comment1#**

“Firstly, we would like to ask you to improve the literature review section of your manuscript by incorporating some relevant papers. For example, Bao and Huang (2021) and Zhou et al. (2022) provide evidence on how unexpected shocks affect small businesses in China, while Huang et al. (2020) investigate the effectiveness of social distancing during the COVID-19 pandemic. These papers are connected to your study as they all examine the impact of external factors on financial markets and may provide useful insights for your analysis. We encourage you to discuss these papers in your literature review and explain how they relate to your research question”

**The author's answer:**Thank you for your advice on the literature review section, under your guidance, we searched the relevant papers and found that external shocks can have a significant impact on financial market. With your help it has improved my research a lot, and we have also added these papers in the literature review section and analyzed them specifically in the article in relation to our research. We have cited these papers as an addition to the literature in this paper, labeling them in the following order (they have been highlighted in red in the Reference section of file “Revised Manuscript with Track Changes”):

[21.Bao Z, Huang D. Shadow Banking in a Crisis: Evidence from Fintech During COVID-19. J Financ Quant Anal. 2021;56(7):2320–55.

22.Zhou Y, Huang D, Chen M, Wang Y, Yang X. How Did Small Business Respond to Unexpected Shocks? Evidence from a Natural Experiment in China. Evidence from a Natural Experiment in China (March 26, 2022). 2022.

23.Huang D. How Effective Is Social Distancing? SSRN Electronic Journal. 2020(6186).

26.Chen M, Huang D, Wu B. Interlocking Directorates and Firm Performance: Evidence from China. Available at SSRN 4005022. 2022.

30.Chen M, Wang Y, Wu B, Huang D. Dynamic Analyses of Contagion Risk and Module Evolution on the SSE A-Shares Market Based on Minimum Information Entropy. Entropy (Basel). 2021;23(4):434.

31.Chen M, Li N, Zheng L, Huang D, Wu B. Dynamic correlation of market connectivity, risk spillover and abnormal volatility in stock price. Physica A. 2022;587:126506.]

We find that citing these papers makes my literature review more complete and comprehensive and enriches the exploration of the related area of the impact of external uncertainty shocks on pan-financial markets.

**Comment2#**

“Secondly, we would like to provide some comments to help you improve your manuscript. Firstly, we suggest that you provide a more detailed explanation of the methodology used in your study, particularly the LASSO-VAR-DY framework. This will help readers better understand your analysis and replicate your results. ”

**The author's answer:** We apologize for the lack of detail in the presentation of the framework, which caused problems for the editor's review process. We recognize the comments you made that the presentation of the LASSO-VAR-DY framework in the previous manuscript was a bit too brief and academic. We have carefully analyzed and revised the presentation of the model in this regard, and have refined this section in the new manuscript. We have explained the model in detail to better serve the journal and readers in analyzing and replicating the results. The Research design section of the uploaded manuscript has been revised in detail and highlighted in red.

**Comment3#**

“ Secondly, we recommend that you provide a more comprehensive discussion of the implications of your findings for policymakers and practitioners. For example, how can your results be used to prevent and mitigate systemic risk in China's pan-financial market? ”

**The author's answer:**We apologize for the incomplete consideration of the policy proposals. We have made relevant changes in the Conclusions and suggestions section of the manuscript. We discuss the implications of the findings of this study for policymakers, firm operators, and investors, and make recommendations specific to each subject. The details as follows: “ Uncertainty brought about by climate shocks exacerbates the trend of the pan-financial market in traditional financial market, profoundly altering the intensity, scope of impact, and path of contagion of systemic financial risks, which provides essential insights into the current systemic financial risks: Firstly,promote cooperation among firms in China’s pan-financial market........Secondly, investors' ability to assess and manage climate risks should be strengthened........Thirdly, the Government should implement differentiated regulation. ”

**Comment4#**

“ Finally, we suggest that you revise the language and structure of your manuscript to improve its clarity and readability. ”

**The author's answer:**We apologize for the poor language of our manuscript. We worked on the manuscript for a long time and the repeated addition and removal of sentences and sections obviously led to poor readability. We have now worked on both language and readability and have also involved native English speakers for language corrections. We really hope that the flow and language level have been substantially improved.

Overall, these are just the revisions we have made. If the academic editors have found some other problems in the literature review change part or think that any part of the revision of this paper still needs to be improved, please write to us again. We can do further communication, in order to look forward to our cooperation more pleasant and our paper more perfect. We will strive to publish this paper in PLOS ONE as soon as possible.

Thank you very much for your attention and time. Look forward to hearing from you.

Yours sincerely,

Kai-wei Jia

10 October 2023

School of Business Administration, Liaoning Technical University, Huludao, Liaoning,China

Te1:86+15104295228

E-mail:jiakaiwei@lntu.edu.cn
