# Supplementary material for: Temperature difference and systemic risk: Evidence from LASSO-VAR-DY based on China’s pan-financial market
Source: PLoS One. 2024 Mar 15;19(3):e0295575. doi: 10.1371/journal.pone.0295575 (PMC10942056; doi:10.1371/journal.pone.0295575)
Supplement: Attachment — Submitted filename: Response to Reviewers.docx [file pone.0295575.s003.docx]

**Response to Reviewers**

Dear Academic Editor,

Thank you very much for your comments and professional advice. These opinions help to improve acade micrigor of our article Based on your suggestion and request, we have made corrected modifications on the revised manuscript. Meanwhile, the manuscript had be reviewed and edited by language and structure. We hope that our work can be improved again. Furthermore,we would like to show the details as follows:

**Reviewers' comments:
Reviewer's Responses to Questions**

**Comments to the Author
Comment#4.** *Have the authors made all data underlying the findings in their manuscript fully available?
The [PLOS Data policy](http://www.plosone.org/static/policies.action#sharing) requires authors to make all data underlying the findings described in their manuscript fully available without restriction, with rare exception (please refer to the Data Availability Statement in the manuscript PDF file). The data should be provided as part of the manuscript or its supporting information, or deposited to a public repository. For example, in addition to summary statistics, the data points behind means, medians and variance measures should be available. If there are restrictions on publicly sharing data—e.g. participant privacy or use of data from a third party—those must be specified.*

*Reviewer #1: No*

**The author's answer:** I have refined the dataset and submitted the data to the system as part of the supporting information. The data behind the mean, median, and variance measures have been included in the latest version of the data.

**Comment#6.** *Review Comments to the Author
Please use the space provided to explain your answers to the questions above. You may also include additional comments for the author, including concerns about dual publication, research ethics, or publication ethics. (Please upload your review as an attachment if it exceeds 20,000 characters)*

**Comment#6.1. Literature Review Improvement:**
*We suggest that you incorporate some relevant papers into your literature review. These papers can help to strengthen the connections between your research and existing literature, as well as provide additional insights into the topic. Specifically, we recommend the following papers:
- Wu, B., Huang, D., & Chen, M. (2023). Estimating contagion mechanism in global equity market with time-zone effect. Financial Management, 52, 543–572.
Li, N., Chen, M., Gao, H., Huang, D., & Yang, X. (2023). Impact of lockdown and government subsidies on rural households at early COVID-19 pandemic in China. China Agricultural Economic Review, 15(1), 109–133.
Li, N., Chen, M., & Huang, D. (2022). How Do Logistics Disruptions Affect Rural Households? Evidence from COVID-19 in China. Sustainability, 15(1), 465.
Yu, D., & Huang, D. (2023a). Cross-sectional uncertainty and expected stock returns. Journal of Empirical Finance, 72, 321–340.
Yu, D., & Huang, D. (2023b). Option-Implied Idiosyncratic Skewness and Expected Returns: Mind the Long Run. Available at SSRN 4323748.
Yu, D., Huang, D., & Chen, L. (2023). Stock return predictability and cyclical movements in valuation ratios. Journal of Empirical Finance, 72, 36–53.
Yu, D., Huang, D., Chen, L., & Li, L. (2023). Forecasting dividend growth: The role of adjusted earnings yield. Economic Modelling, 120, 106188.
These papers can provide valuable insights into the dynamics of financial markets, risk spillover, and the impact of external factors on market stability. For example, Bao and Huang (2021) investigate the role of shadow banking during a crisis, which can be relevant to your discussion on systemic risk in the financial sector. Chen et al. (2022a) and Wu et al. (2023) both explore the dynamics of market connectivity and risk spillover, which can help to contextualize your findings on the impact of temperature differences on systemic risk. Additionally, Chen et al. (2022b) examine the relationship between interlocking directorates and firm performance, which can provide insights into the potential channels through which temperature differences may affect systemic risk.*

**The author's answer:** 1. Thank you for your advice on the literature review section. Under your guidance, we searched the relevant papers. We found that external shocks can significantly impact the financial market. With your help, it has dramatically improved my research. We have also added these papers in the literature review section and analyzed them specifically in the article concerning our study. We have cited these papers as an addition to the literature in this paper, labeling them in the following order (they have been highlighted in red in the Reference section of file "Revised Manuscript with Track Changes").2. We also reorganized the literature, collected authoritative literature in related fields and re-improved the literature review by combining with the research topic of this paper, such as Journal of Climate Finance, Energy Economics, and other journals, which enriched the impact of financial institutions' micro, macroeconomic, and uncertainty shocks on systemic risk (these are reflected in the section of the paper "Literature review of the impact of internal factors, external shocks and financial market factors on systemic risk" section). These, combined with your suggestions and guidance, will make my literature review more comprehensive and relevant.

[25.Bao Z, Huang D. Shadow Banking in a Crisis: Evidence from Fintech During COVID-19. Journal of Financial and Quantitative Analysis. 2021;56.

26.Zhou Y, Huang D, Chen M, Wang Y, Yang X. How Did Small Business Respond to Unexpected Shocks? Evidence from a Natural Experiment in China. Evidence from a Natural Experiment in China (March 26, 2022). 2022.

27.Huang D. How Effective Is Social Distancing? SSRN Electronic Journal. 2020(6186).

32.Yang Z, Chen Y, Huang Z. Research on Influencing Factors and Transmission Channels of Systemic Risks under International Shocks.Economic Research Journal.2023,58(01):90-106.

37.Chen M, Huang D, Wu B. Interlocking Directorates and Firm Performance: Evidence from China. Available at SSRN 4005022. 2022.

39.Chen M, Wang Y, Wu B, Huang D. Dynamic Analyses of Contagion Risk and Module Evolution on the SSE A-Shares Market Based on Minimum Information Entropy. Entropy (Basel). 2021;23(4):434.

40.Chen M, Li N, Zheng L, Huang D, Wu B. Dynamic correlation of market connectivity, risk spillover and abnormal volatility in stock price. Physica A. 2022;587:126506.

41.Wu B, Chen M, Huang D. Estimating Contagion Mechanism in Global Equity Market with Time-Zone Effects. Social Science Electronic Publishing.]

**Comment#6. 2. Detailed Comments and Suggestions:
Comment#6.2.1-** Please provide a more detailed explanation of the LASSO-VAR-DY framework and its advantages in analyzing the impact of temperature differences on systemic risk. This will help readers to better understand the methodology and its relevance to your research question.

**The author's answer:** We apologize for the lack of detail in the presentation of the framework, which caused problems in the editor's review process. We recognize your comments that the production of the LASSO-VAR-DY framework in the previous manuscript needed to be longer and more academic. We have refined the section "Research design" and adjusted the headings of each subsection appropriately. In the section "Construction of a pan-financial market volatility spillover network based on the LASSO-VAR-DY framework," we explain the advantages of the LASSO-VAR-DY framework in analyzing the impact of temperature differentials on pan-financial systemic risk. The risk spillover formula is supplemented in the "DY spillover index" section. At the same time, we have carefully analyzed and revised the model's overall presentation and refined this section in the new manuscript. We also provide detailed explanations of the specific settings of the model parameters in the empirical section to better serve the journal and readers in analyzing and replicating the results. The study design section of the uploaded manuscript has been revised and highlighted in red.

**Comment#6.2.2-** *Consider conducting additional robustness checks to validate your findings. For example, you could explore alternative measures of temperature differences or systemic risk, or test the sensitivity of your results to different model specifications.*

**The author's answer:** Thanks to the reviewers' suggestions for refining robustness, additional robustness tests were performed in this paper. The thesis uses six methods to test the robustness of the model. Firstly, in the regression analysis, the variables are deflated by 5% to consider the presence of extreme values in the data. Second, regarding the chance of sample time selection, the regression analysis is chosen to examine the robustness of the results for the sample from 2014 to 2021. Third, considering the effect of variable selection, the monthly average of temperature 2 meters above the surface (Temperature_2m) from the same database was selected to replace the core independent variable for the regression analysis. Fourth, considering the alternative measures of systematic risk of the explanatory variables, the choice of replacement of the explanatory variables selected the centrality of network topology features. This means that the Degree variable replaced the centrality of feature vectors for regression. Fifth, considering the sensitivity of the test results to different model scales, this paper resets the thresholds of network filtering. It selects 0.3 and 0.5 quartile thresholds to extract adequate information on the network obtained by Pearson's correlation coefficient. These six robustness tests, which consider alternative measures of temperature difference and systematic risk, are combined with the reviewers' suggestions to assess the sensitivity of the model specification to allow a robust demonstration of the empirical results.

**Comment#6.2.3-** *In your discussion of the results, please provide more context on the potential mechanisms through which temperature differences may affect systemic risk. This will help to strengthen the theoretical underpinnings of your findings and provide a more comprehensive understanding of the relationship between climate change and financial stability.*

**The author's answer:** Thanks to the reviewer's suggestion that the previous manuscript's results discussion section lacked background information on the potential mechanisms of temperature difference on pan-financial systemic risk, I have refined the relevant theoretical background in the empirical results discussion section of the latest version of the manuscript. In the area "Empirical analysis of the mechanisms influencing systemic risk in the pan-financial market," several subsections are devoted to the refinement of the background information on the mechanisms, the mechanism of temperature difference on systemic risk, the means of higher and colder temperature on systemic risk in the pan-financial market, and the theoretical mechanism of temperature difference on systemic risk in the subsector. In the section "Identifying potential pathways for the transmission of the temperature difference and systemic risk in the pan-financial market," the relevant theoretical background has also been added, and further refinements have been made for the contagion path of the minimum spanning tree and the dynamic evolutionary mechanism of temperature difference on systemic risk. These modifications are marked in red font in the trace manuscript..

**Comment#6.2.4-** *It would be helpful to include a discussion of the policy implications of your findings. For example, what do your results suggest about the potential role of climate change mitigation policies in reducing systemic risk in the financial sector?*

**The author's answer:** We apologize for the incomplete consideration of the policy proposals. We have made relevant changes in the Conclusions and Suggestions section of the manuscript. We discuss the implications of the findings of this study for policymakers, firm operators, and investors and make recommendations specific to each subject. The details are :[ First, promote cooperation among firms in pan-financial markets....... Second, it strengthens investors' ability to assess and manage climate risk....... Third, policymakers should consider climate change factors.......Fourthly, Governments should expand the scenarios for monetary policy and macroprudential regulation.......].

We apologize for the poor language of our manuscript. We worked on the manuscript for a long time, and the repeated addition and removal of sentences and sections led to poor readability. We have now worked on language and readability and have also involved native English speakers for language corrections. We hope that the flow and language level have been substantially improved.

Overall, these are just the revisions we have made. If the academic editors have found other problems in the literature review or think that any part of the revision of this paper still needs to be improved, please write to us again. We can do further communication to look forward to our cooperation more pleasant and our article more perfect. We will strive to publish this paper in PLOS ONE as soon as possible.

Thank you very much for your attention and time. I look forward to hearing from you.

Yours sincerely,

Kai-wei Jia

22 November 2023

School of Business Administration, Liaoning Technical University, Huludao, Liaoning,China

Te1:86+15104295228

E-mail:jiakaiwei@lntu.edu.cn
