# Supplementary material for: Temperature difference and systemic risk: Evidence from LASSO-VAR-DY based on China’s pan-financial market
Source: PLoS One. 2024 Mar 15;19(3):e0295575. doi: 10.1371/journal.pone.0295575 (PMC10942056; doi:10.1371/journal.pone.0295575)
Supplement: Attachment — Submitted filename: Response to PLOS ONE.docx [file pone.0295575.s004.docx]

Response to PLOS ONE

To:	"PLOS ONE" plosone@plos.org

From:	"Kaiwei Jia " jiakaiwei@lntu.edu.cn

Subject:	Temperature difference and systemic risk:

Evidence from LASSO-VAR-DY based on China's pan-financial market

Dear PLOS ONE :

Thank you very much for your comments and professional advice. These opinions help to improve the professionalism and standardization of my article. Based on your suggestion and requirements for the PLOS ONE journal, I have made corrected modifications to the manuscript. My changes to the manuscript are as follows: I have uploaded my manuscript and figures. Changes have been red-flagged in the Revised Manuscript with Track Changes file. The specific modifications are as follows:

Comment#1.General remarks :1. The note is about causality. In his work, the author very often uses the term "impact" when he uses only regression-type methodology. The correct solution is to use methods based on the concepts of impact effects. The use of panel econometrics methods is a good substitute choice in the absence of counterfactual analyses. It is best to use the terms "relationship", "relationship", "association".

The author's answer#1:First of all, I am very grateful to the reviewer for his suggestions regarding the use of regression methods, causality, and the term impact in this paper. I have carefully considered the term "relationship," which is indeed an excellent alternative to using panel econometric methods in the absence of counterfactual analysis. The term "impact" in this paper draws on relevant papers in the area of climate change and financial stability.

[reference19.Chabot M, Bertrand JL. Climate risks and financial stability: Evidence from the European financial system. J Financial Stab. 2023;69:101190.]. In this paper, the author estimates the influence of physical and transition risks on the European financial system through bank-level and system-wide measures of financial stability. They find that Scope 3 greenhouse gas emissions and chronic and acute climate risks negatively affect financial stability at both the financial institution and system levels. Temperature anomalies, heat waves, wildfires, and droughts are among the most significant risks.”Also, in this paper, the author points out that the influence of climate risks on financial stability is carried out using panel data models. A recent literature review on statistical approaches applied to climate and economics showed that the use of panel data is highly relevant when the research question aims to understand the response of a system as a whole to climate change.

[reference46.Song XN, Fang T. Temperature shocks and bank systemic risk: Evidence from China. Finance Research Letters. 2023;51.https://doi.org/10.1016/j.frl.2022.103447 ]. In this paper, the authors investigate the impact of temperature shocks on the systemic risk of Chinese listed banks, which is measured by a new nonlinear tail-event driven network (TENET) conditional value-at-risk (CoVaR) under expectation. We find that higher temperatures significantly increase the bank's systemic risk, and the impact of temperature shocks is significantly more significant during colder periods.

The areas of climate risk and financial stability examined in this paper are consistent with these papers, and both use panel regressions to examine the impact relationship between the two. Thus, the paper draws on the term "impact" used by these scholars in the discussion section of the main text.

Comment#2.General remarks :2. The note concerns the statistical significance of variables in regression models, to which the author attaches great importance and (also) on their basis verifies his hypotheses. In empirical studies, it often happens that statistical significance is not that important. I don't think it's defective. The thing fits into the current debate of scientists from various fields (excessive faith in p-value).

The author's answer#2:Thank you for the advice you have given. I also agree with you that empirical research should still be based on theoretical analysis. After all, the results of the empirical analysis are only support for the theoretical analysis. Thus, my selection of the topic is established on the basis of the theory rather than relying solely on the statistical significance. At the same time, the empirical results in this paper are all based on the software operation directly derived, accurate, and reliable. However, we also refer to the authors' viewpoints and make appropriate standardization and modifications for the expression of statements in the article.

Comment#3.General remarks :3. A flowchart should be added to the article to show the research methodology. Applied models, e.g. FE without any diagnostics. In addition, it is worth considering dynamic processes, i.e. the use of a model, e.g. GMM (The role of trade credit in business operations, Argumenta Oeconomica 2 (37), 189-231).
Additionally, in one FE regression you cannot use time variables (effect of time) and additional macroeconomic variables such as CPI, RATE, etc. The time effect includes all macro variables anyway..

The author's answer#3:On the first point, thank you for your suggestion to make a flowchart of the research methodology and add it to the paper in order to make the presentation of the research methodology more intuitive.

***Fig 1. Research Methods***

On the second point, thank you for providing the suggestion to consider dynamic processes in this paper. Since in the panel two-way fixed effects sector, the panel examined in this paper is a static panel (which means that the lagged terms of the explanatory variables are not included in the explanatory variables). Observing the linear relationship between higher and colder temperature and pan-financial systemic risk but failing to observe the dynamic evolution of the three variables may result in an unclear and ill-defined chain of causal logic. Therefore, in the section "Results and analysis of the dynamic evolutionary patterns of temperature difference and the systemic risk in pan-financial market," we choose to construct a panel vector autoregression model (PVAR) to analyze the systemic risk in pan-financial market and conduct an impulse response (IRF) analysis, so as to more intuitively portray the trend of the impact of temperature difference and the systemic risk in pan-financial market, and to study the dynamic time-varying patterns of evolution of the temperature difference and the systemic risk in pan-financial market.

GMM estimation is not used in this paper for the following reasons:

For GMM estimation, it is mainly used as a method to solve the endogeneity problem, which arises for three main reasons:(i) Omission of variables: for example, when studying the influencing factors of wages, the explanatory variables include years of education, but personal competence is not included due to the limitations of the measurement of the indicator, thus resulting in the omission of the influencing factors. (ii) Measurement error of variables: for example, we are always accustomed to using students' test scores to measure IQ, but this refers to the bias. A person's IQ can not be expressed simply through grades; a person's grades will be affected by other uncertainties; this way of measurement is too simple and refers to error. (iii) Bidirectional causality: for example, years of education affect wages.

On the one hand, The core explanatory variable used in this paper is "temperature difference," and the explanatory variable is "systematic risk." The temperature difference is a strictly exogenous variable compared with systematic risk. Most importantly, the judgment of endogeneity should be based on theoretical analysis, and the use of a series of Stata verifications only plays an auxiliary role. We generally will not be entirely in accordance with the state test to determine the existence of endogeneity.

[Form for endogeneity test]

***Table . Endogeneity test.***

|  | **Dependent variable: systemic risk of pan-financial market** |
| --- | --- |
| Temp40 | 0.4955 |
| Temp80 | 0.5937 |
| Temp120 | 0.5539 |
| Temp_plus40 | 0.1697 |
| Temp_minus40 | 0.1587 |
| Temp_plus80 | 0.2272 |
| Temp_minus80 | 0.1732 |
| Temp_plus120 | 0.2051 |
| Temp_minus120 | 0.1859 |

Note:Results are p-values for endogeneity tests.

The table shows the results of the endogeneity test for each variable as a core explanatory variable, which means that the original hypothesis is not rejected when the p-value is more significant than 0.05, proving that the model is not endogenous.

On the other hand, there is no bidirectional causality because only the temperature difference affects systemic risk, which does not affect changes in temperature. Finally, there may be an omitted variable problem, which is also considered in this paper, which uses a two-way fixed-effects model, with individual-fixed effects controlling for the inherent characteristics of firms that do not change over time and time-fixed effects controlling for the time trend of systemic risk.

On the third point, thank you for your suggestion that "Additionally, in one FE regression, you cannot use time variables (effect of time) and additional macroeconomic variables such as CPI, RATE, etc. The time effect includes all macro variables anyway."We have reconsidered this issue and responded to it. In the first column of the robustness test in Table 6, re-doing the two-way fixed effects after removing the macro control variables. The results show that the effects of the variables are consistent with the original results. However, the goodness of fit is smaller than the original results, but this does not affect the robustness of the results. To this, we would like to give the following explanation.

Analyzing empirical papers from an econometric writing perspective, this type of research methodology is also evidenced in many journal papers. For example, [1]Wang D, Wang Z, Cai W, et al. Digital inclusive finance, higher education expansion and regional carbon emissions:evidence from China[J]. International Review of Economics & Finance, 2024, 89: 1091-1101.[2]Chen Z, Huang W, Zheng X. The decline in energy intensity: does financial development matter?[J]. Energy Policy, 2019, 134: 110945.

And references cited in my paper [reference54.Aevoae GM, Andrieș AM, Ongena S, Sprincean N. ESG and systemic risk. Appl Econ. 2022;55(27):3085–109.https://doi.org/10.1080/00036846.2022.2108752.]. In this paper, the author used the control variables at the banking system level (Bank Concentration), country-level characteristics (Real GDP Growth and Inflation), and the overall level of governance in a country. Using a panel model that accounts for individual and time effects, the author documents a beneficial impact of the ESG Combined Score and Governance pillar on banks’ contribution to system-wide distress, analyzing a panel of 367 publicly listed banks from 47 countries over the period 2007–2020. The findings stress the importance of integrating banks’ ESG disclosure into regulatory authorities’ supervisory mechanisms as qualitative information.

Analyzed from a theoretical perspective, macroeconomic control variables and time effects in two-way fixed effects have different effects. Macroeconomic control variables are historical data, such as CPI, GDP, China Real Estate Climate Index, etc., and each company's stock volatility is closely related to a certain extent. It will have an impact on the role that must be addressed. At the same time, the historical data used for macro control variables may be lagged and noisy, which means that some unexpected events cannot be captured instantly. For example, in the 2008 economic crisis, real estate collapsed. However, macroeconomic variables were performing well at the time. When Lehman Brothers collapsed, the macroeconomic bank balance sheets in the United States could not reflect this sudden event, which means that time-fixed effects need to be incorporated to overcome these shocks to some extent.

Thus, this regression approach of incorporating two-way fixed effects of micro-level and macro-level control variables is also the result of our synthesis from both theoretical analysis and empirical measurement perspectives.

In addition to this, this paper also reorganizes the literature review section, adding to it the latest literature related to this topic.

In summary, here is my explanation for the fund change. If you have found other problems in the article or think that any part of the revision of this paper still needs to be improved, please write to us again. We can do further communication to look forward to our cooperation being more pleasant and our article being more perfect. We will strive to publish this paper in PLOS ONE as soon as possible.

Thank you very much for your attention and time. I look forward to hearing from you.

Yours sincerely,

Kai-wei Jia

13 February 2024

School of Business Administration, Liaoning Technical University, Huludao, Liaoning,China

Te1:86+15104295228

E-mail:jiakaiwei@lntu.edu.cn
